# Diffusion of innovation in radiation oncology in the United States

**DOI:** 10.1259/bjro.20200025

**Published:** 2020-08-26

**Authors:** Patricia Sansourekidou, Vasileios Margaritis, Wen-Hung Kuo

**Affiliations:** 1 Department of Radiation Oncology, Montefiore Health System - White Plains Hospital Center for Cancer Care, White Plains, NY, 10601, United States; 2 Walden University, Minneapolis, MN, United States

## Abstract

**Objective:**

To develop an instrument for quantifying innovation and assess the diffusion of innovation in radiation oncology (RO) in the United States.

**Methods:**

Primary data were collected for using total population convenience sampling. Innovation Score and Innovation Utilization Score were determined using 20 indicators. 240 medical physicists (MPs) practicing in RO in the United States completed a custom Internet-based survey.

**Results:**

Centers with no academic affiliation are trailing behind in innovation in total (MD = 1.65, 95% C I[0.38,2.917], *p* = 0.011, *d* = 0.351), in patient treatment (MD = 0.39, 95% CI [0.021,0.76], *p* = 0.038, *d* = 0.282), and workflow innovation (MD = 7.09, 95% CI [0.78,13.39], *p* = 0.028, *d* = 0.330). Centers with no academic affiliation are trailing behind in innovation utilization in total (MD = 0.46, 95% CI [0.05,0.86], *p* = 0.028, *d* = 0.188). Rural center are trailing behind in patient positioning in innovation (MD = 0.31, 95% CI [0.011,0.612], *p* = 0.042, *d* = 0.293) and innovation utilization (MD = 16.22, 95% CI [0.73,31.72], *p* = 0.04, *d =* 0.608). Rural centers are trailing behind in innovative treatments (MD = 0.62, 95% CI [0.23,1.00], *p* = 0.002, *d* = 0.457). Motivation (r_s_ = 0.224, *p* = 0.002) and appreciation (r_s_ = 0.215, *p* = 0.003) were statistically significant personal factors influencing innovation utilization.

**Conclusions:**

There is a wide range of innovation across RO centers in the United States. RO centers in the United States are not practicing as innovative as reasonably achievable.

**Advances in knowledge:**

This work quantified how innovative RO in the United States is and results provide guidance on how to improve it in the future.

## Introduction

The field of radiation oncology (RO) has experienced rapid growth in the past 25 years, with technological advancement as the driving force. The implementation of innovative methods in healthcare and how these methods reach the general population is an active field of research.^1,2^ Available data specific to RO and an instrument to effectively measure the diffusion of innovation in the field are lacking.

Jacobs et al^3^ applied the Delphi method to determine indicators for innovation in RO by creating consensus guidelines among RO chairpersons to define innovation in RO and used semi-structured interviews across 15 RO centers in the Netherlands. The authors derived 13 indicators in 4 categories, indicators in 4 categories, as described in Table 1. These indicators are a benchmark in an attempt to study innovation in RO. Innovation has been successfully quantified in the Netherlands with a small number of centers. Dutch centers implement on average 12 innovations per year (range 5–25).^4^ The average number is sufficiently large and the authors concluded that Dutch radiotherapy centers are highly accepting of innovation.

**Table 1.** Overview of radiation oncology-related innovation indicators developed by Jacobs et al.^3^

Category	Indicator
Product innovation	Number of introductions of new or significantly improved treatments new to radiotherapy or new to the clinic Number of new positioning devices for patient treatment Number of approved patents Percentage of patients in Phase III randomized trials approved by Institutional Review Board Percentage of patients in Phase I-II trials approved by Institutional Review Board
Technological innovation	Frequency of implementation of new medical devices Number of products for which royalties have been obtained or which have been sold to the industry Number of Conformité Européenne marked products that have been produced by the department
Market innovation	Percentage of patients from outside the market area Number and percentage of new general hospitals that refer the desired patient population
Organizational innovation	New practices for organizing procedures New methods for organizing work responsibilities and decision making New methods for organizing external relationships with other organizations or public institutions

Similar studies of innovation in the United States were previously lacking. The purpose of this study was to develop an instrument to measure the diffusion of innovation in RO, measure the diffusion of innovation in RO in the United States, and assess possible diffusion patterns. In this study, the problem of diffusion of innovation was addressed from the point of view of medical physicists (MPs), who are responsible for the acceptance, commissioning, and implementation of innovative techniques in RO. In RO, there are fragmented data on innovation and there has been limited analysis of the barriers to developing and implementing new technology in RO.^5^ This study is the first to systematically quantify the diffusion of innovation in RO in the United States.

## Methods and materials

Studies attempting to define and measure innovation in RO rely on custom survey design, as there is no centralized reporting mechanism.^3,4^ A custom survey instrument was developed in order to conduct this analysis, largely based on the results from Jacobs et al..^3^ SurveyMonkey^6^ was used as the platform to develop the internet-based custom survey content, available in Supplementary Material 1. The survey contained a total of 70 questions and took approximately 5–10 min to complete. The survey included questions on multiple demographic and practice information, followed by the innovation indicators in Table 2. A comment section was available for open-ended feedback after the quantitative section. At the end of the survey, respondents were asked to enter an email address if they wish to receive a $10 Amazon gift card as an incentive to participate.

Supplementary Material 1.

**Table 2.** Indicators used for innovation score determination

Category	Indicator
Patient positioning	Surface guided radiation therapy Respiratory gating Breath-hold
Patient treatment	Stereotactic body radiosurgery Stereotactic cranial radiosurgery Robotic radiosurgery Intraoperative radiation therapy Flattening free beams
Treatment planning	Automatic contouring Deformable image registration Automatic planning Adaptive planning
Quality assurance	Portal dosimetry Trending Automatic QA Automatic plan checks
Workflow	Clinical trials New procedures New responsibilities New external relations

Face validity and content validity were demonstrated by an expert panel. The expert panel was used to assess edits from Jacobs et al^3^ to apply to the United States, as well as clarity of wording, applicability of answers, etc. Five MPs with expertise on the subject were contacted in November 2018 and asked to review the survey and identify any ambiguity in the wording of the questions. Each expert panelist was contacted via email and sent a preliminary version of the survey. Feedback was requested in writing within a week. Comments were received during a 2-week period. Comment examples included the anonymity of the survey, stratification techniques, and length of the consent form. Additionally, comments were requested from the American Association of Physicists in Medicine (AAPM) Technology Assessment Office and Corporate Advisory Board. Four blinded field experts reviewed the survey and provided feedback on the structure and levels of measurement. All comments were used to improve the final survey questions before deploying the study to the target population and enhance the study’s validity.

Primary data were collected for this study using total population convenience sampling. The MedPhysUSA listserv and AAPM Blackboard were used to recruit respondents. Respondents were contacted passively by using the forums mentioned above. The invitation contained a brief description, incentive information, and a link to participate. The survey was conducted between April 27 and June 1, 2019. Two reminders were sent approximately 10 days apart. The survey remained open for a total of 35 days. Response rate calculations are not applicable to this study, as respondents were contacted passively through open forums.

**Figure 1.**
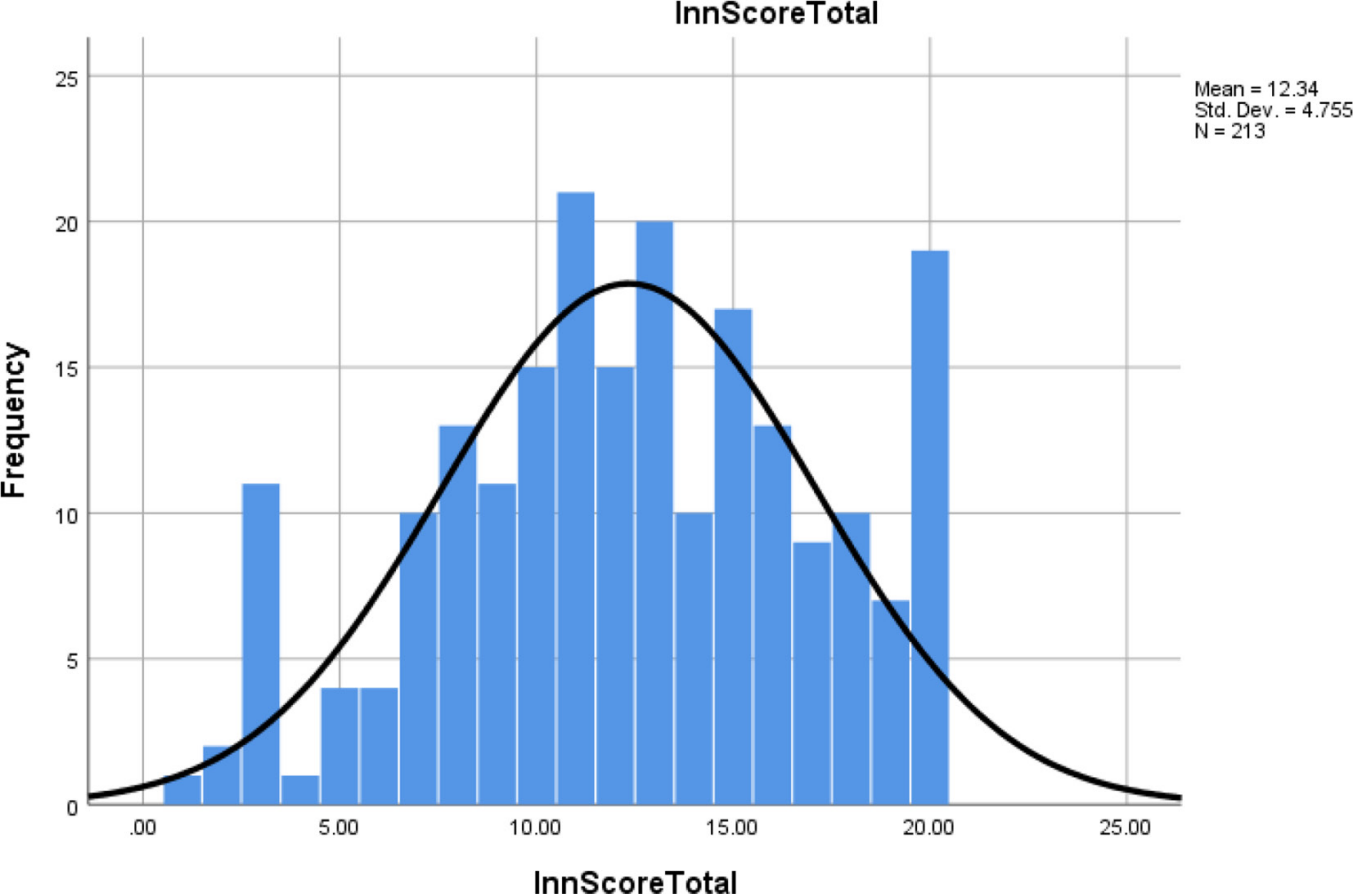
Distribution of RO innovation score.

The dependent variable, innovation score, was measured using 20 unique indicators in five categories as shown in Table 2. Participant answers were entered using a slider with scale 0–100. For values entered as 0 or 1 on the slider, it was assumed that the respondents meant to not move the slider at all and that the type of innovation was not available. For values entered as 2–100 on the sliding scale, it was assumed that the respondents had the technology available to them. This categorized each respondent as having or not having the innovation. The innovation indicators were summed to calculate the innovation score for each category and the total innovation score for each respondent. This provided a measure for the diffusion of available innovative techniques and will be subsequently referred to as *innovation score*. The innovation score distribution is shown in Figure 1, and it appears to be normally distributed.

**Figure 2.**
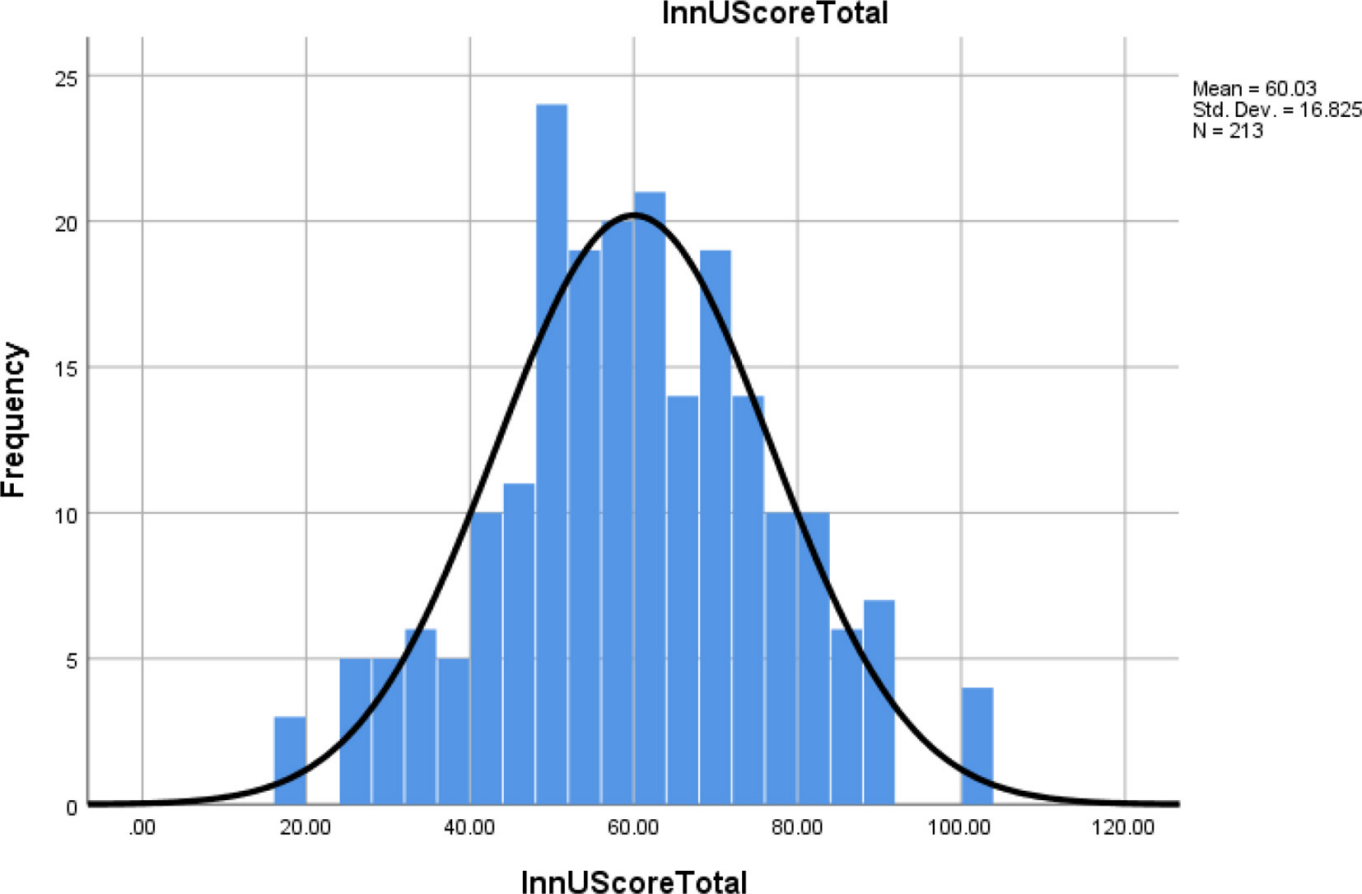
Distribution of RO innovation utilization score.

Further, the exact number on the scale of individual responses was used to assess how respondents actually used the innovations available to them. This will be subsequently referred to as *innovation utilization score*, and it is distinctly different from the previously defined innovation score. A similar method described for innovation score was used for the innovation utilization score. The mean of the responses in each of the indicators in each category was used to calculate the innovation utilization score in each category separately. The innovation utilization score was calculated by adding the innovation utilization score in the five categories. The innovation utilization score distribution is shown in Figure 2, and it appears to be normally distributed.

The independent variables are described in detail in Table 3. University affiliation was measured as a binary yes or no. Zip code text entry was converted to categorical using the RUCA continuum,^7^ described in Supplementary Material 2. Gender was binary male or female. Age was measured as a continuous variable and was recoded into categorical. Education was measured as Master’s, Doctoral or other. Residency status was measured as a categorical variable (yes, no, no didn’t need). ABR status was measured as yes, yes/other, or no. Interpersonal channels were measured as a continuous variable, using the number of meetings attended. Organizational structure was measured as a categorical variable as physicist, physician, administrator. Group size was measured as a categorical variable based on the number of physicists in the practice. Opinion leadership was measured as a binary variable as yes or no. Appreciation and motivation were measured as continuous variables. The operationalization of appreciation and motivation was based largely on prior published studies in other healthcare professionals.^8,9^

Supplementary Material 2.

**Table 3.** Operationalization of constructs

	Variable	Level of measurement
Dependent	Innovation score	Continuous
Independent	University affiliation	Binary
	Zip code	Categorical
	Gender	Categorical
	Age	Continuous
	Degree	Binary
	Residency	Categorical
	ABR status	Categorical
	Interpersonal channels	Continuous
	Organizational structure	Categorical
	Group size	Categorical
	Opinion leadership	Categorical
	Appreciation	Continuous
	Motivation	Continuous

Incomplete data entries were excluded pairwise when applicable. The analysis was conducted using SPSS 25. Cohen’s *d* effect size was calculated manually where applicable. Construct validity was demonstrated using principal component analysis after data collection, and the overall Kaiser–Meyer–Olkin measure was 0.634. The scale had a high level of internal consistency, as determined by a Cronbach's α of 0.963 using interitem reliability and split-half method, derived after data collection.

The Shapiro–Wilk test for normality was performed for innovation score and innovation utilization score, using university affiliation and urbanicity as factors. The results of the Shapiro–Wilk test were not statistically significant for the innovation score (*p* = 0.611) and for the innovation utilization score (*p* = 0.699). Thus, the data were normally distributed. The Q–Q plots were also normal. Parametric *t*-test was subsequently used for both university affiliation and urbanicity.

An independent *t*-test was performed to determine if there were differences in innovation score between university and non-university centers for the total innovation score and for the innovation utilization score. There were no outliers in the data, as assessed by inspection of a boxplot. There was homogeneity of variances, as assessed by Levene’s test for equality of variances for both the total innovation score (*p* = 0.689) and the innovation utilization score (*p* = 0.129). An independent *t*-test was performed to determine if there were differences in innovation score between metropolitan and non-metropolitan centers for the total innovation score and for the innovation utilization score. There were no outliers in the data, as assessed by inspection of a boxplot. There was homogeneity of variances, as assessed by Levene’s test for equality of variances for both the total innovation score (*p* = 0.478) and the innovation utilization score (*p* = 0.855). It is noted that total innovation score was assessed using binary RUCA categorization 1 and 2–9, while innovation utilization score was assessed using binary RUCA categorization 1–3 and 4–9. For metropolitan and non-metropolitan centers, a qualitative analysis was also performed, using a heatmap. The population was superimposed with innovation score (darker green, higher population). Additionally, all RO centers that are currently operational in the United States are superimposed as black squares.^10^

Bivariate correlation analysis was performed for the continuous variables: appreciation, motivation and number of meetings attended. Shapiro–Wilk test for normality was performed for innovation score and innovation utilization score using appreciation, motivation, and number of meetings as factors (continuous variables). The results of the Shapiro–Wilk test were statistically significant for all three parameters (*p* < 0.001). Thus, the data are not normally distributed. The Q–Q plots were also not normal. Spearman correlation was used to perform bivariate analysis for appreciation and motivation.

Bivariate analysis was performed for binary variables: gender, opinion leadership, education and residency status. Bivariate analysis was performed for categorical variables: age, DABR status, organizational structure and group size.

## Results

At the survey closure, 265 responses were collected. 25 responses contained no answers. The final sample size was *N* = 240. Statistically significant results are presented in Tables 4 and 5. The mean innovation score difference for centers with university affiliation (*M =* 13.19, SD *=* 4.76) is higher than the mean innovation score for centers without a university affiliation (*M =* 11.55, SD *=* 4.63), a statistically significant difference MD = 1.65, 95% CI [0.38,2.917], *t*(211) = 2.56, *p* = 0.011, *d* = 0.351. Additionally, the patient treatment innovation score for university centers (*M =* 3.04, SD *=* 1.43) is higher than the patient treatment innovation score for non-university centers (*M* = 2.64, SD *=* 1.34), a statistically significant difference MD = 0.39, 95% CI [0.021,0.76], *t*(217) = 2.083, *p* = 0.038, *d* = 0.282; the workflow innovation score for university centers (*M =* 2.96, SD *=* 1.51) is higher than the workflow innovation score for non-university centers (*M =* 2.50, SD *=* 1.55), a statistically significant difference MD = 0.46, 95% CI [0.05,0.86], *t*(217) = 2.217, *p* = 0.028, *d* = 0.188. The mean innovation utilization score for centers with university affiliation (*M =* 59.39, SD *=* 17.74) is similar to the mean innovation utilization score for centers without a university affiliation (*M =* 60.62, SD *=* 15.97). The innovation utilization score difference is not statistically significant based on university affiliation. However, for the five categories measured, the mean workflow innovation utilization score for centers with university affiliation (*M =* 54.05, SD *=* 22.85) is higher than the mean workflow innovation utilization score for centers without a university affiliation (*M =* 46.95, SD *=* 19.92), a statistically significant difference MD = 7.09, 95% CI [0.78,13.39], *t*(178) = 2.217, *p* = 0.028, *d* = 0.330. Results did not differ after controlling for metropolitan location.

**Table 4.** Independent Samples *t*-test for RO center innovation score, university affiliation and urbanicity

Category		F	*p*	t	df	*p*	MD	SED	95% CI	
									LL	UL
University affiliation	Total	.160	.689	2.562	211	.**011**	1.649	.644	.379	2.917
	Patient positioning	1.009	.316	1.390	217	.166	.199	.144	−.083	.485
	Patient treatment	.003	.953	2.083	217	.**038**	.392	.188	.021	.762
	Treatment planning	1.510	.221	.937	217	.350	.177	.189	−.196	.550
	QA	1.966	.162	1.758	217	.080	.356	.202	−.043	.754
	Workflow	1.152	.284	2.217	217	.**028**	.458	.207	.051	.865
Urbanicity	Total	.505	.478	1.849	197	.066	1.243	.672	−.083	2.568
	Patient positioning	1.090	.298	2.043	203	.**042**	.314	.154	.011	.618
	Patient treatment	.176	.675	3.145	203	.**002**	.618	.196	.230	1.005
	Treatment planning	.536	.465	1.784	203	.076	.354	.198	−.037	.745
	QA	1.997	.159	.043	203	.966	.009	.216	−.416	.434
	Workflow	3.040	.083	.532	203	.595	.116	.219	−.315	.548

CI, confidence interval.

**Table 5.** Independent samples *t*-test for RO center innovation utilization score, university affiliation and urbanicity

Category		F	*p*	t	df	*p*	MD	SED	95% CI	
									LL	UL
University affiliation	Total	2.317	.129	−.531	211	.596	−1.22	2.311	−5.782	3.33
	Patient positioning	.172	.679	−.822	186	.412	−2.92	3.557	−9.941	4.095
	Patient treatment	.588	.444	−.162	196	.872	−.575	3.546	−7.573	6.423
	Treatment planning	.269	.605	−.944	184	.346	−3.55	3.754	−10.95	3.861
	QA	3.361	.069	−1.38	169	.170	−5.54	4.020	−13.481	2.392
	Workflow	2.542	.113	2.217	178	.**028**	7.086	3.196	.771	13.39
Urbanicity	Total	.034	.855	1.115	197	.266	5.31	4.762	−4.1	14.70
	Patient positioning	1.463	.228	2.067	173	.**040**	16.22	7.849	.732	31.72
	Patient treatment	.067	.795	.698	184	.486	5.13	7.339	−9.35	19.61
	Treatment planning	.221	.639	.829	175	.408	6.56	7.915	−9.06	22.19
	QA	.043	.836	−1.50	161	.135	−12.2	8.098	−28.1	3.84
	Workflow	.214	.644	1.379	170	.170	10.86	7.875	−4.69	26.40

CI, confidence interval; RO, radiation oncology.

Statistically significant results are presented in Tables 4 and 5.The mean innovation score for metropolitan centers (*M =* 12.94, SD *= 4.65*) is similar to the mean innovation score for non-metropolitan centers (*M = 11.69*, SD *=* 4.37). The innovation score difference is not statistically significant based on metropolitan or non-metropolitan status, even though *d* = 0.275. However, for the five categories measured, the mean patient positioning innovation score for metropolitan centers (*M =* 2.21, SD *=* 1.02) is higher than the mean patient positioning innovation score for non-metropolitan centers (*M =* 1.89, SD *=* 1.12), a statistically significant difference MD = 0.31, 95% CI[0.011,0.612], *t*(203) = 2.043, *p* = 0.042, *d* = 0.293. Additionally, the mean patient treatment innovation score for metropolitan centers (*M =* 3.08, SD *=* 1.36) is higher than the mean patient treatment innovation score for non-metropolitan centers (*M =* 2.47, SD *=* 1.33), a statistically significant difference MD = 0.62, 95% CI [0.23,1.00], *t*(203) = 3.145, *p* = 0.002, *d* = 0.457. The mean innovation utilization score for metropolitan centers (*M =* 60.73, SD *=* 16.67) is similar to the mean innovation utilization score for non-metropolitan centers (*M = 55.41*, SD = 15.38). The total innovation utilization score difference is not statistically significant based on metropolitan or non-metropolitan status, even though *d* = 0.331. However, for the five categories measured, the mean patient positioning innovation utilization score for metropolitan centers (*M =* 63.96, SD *=* 23.78) is higher than the mean patient positioning innovation utilization score for non-metropolitan centers (*M =* 47.73, SD *= 29.28*), a statistically significant difference MD = 16.22, 95% CI [0.73,31.72], *t*(173) = 2.067, *p* = 0.04, *d =* 0.608. Results did not differ after controlling for university affiliation.

**Figure 3.**
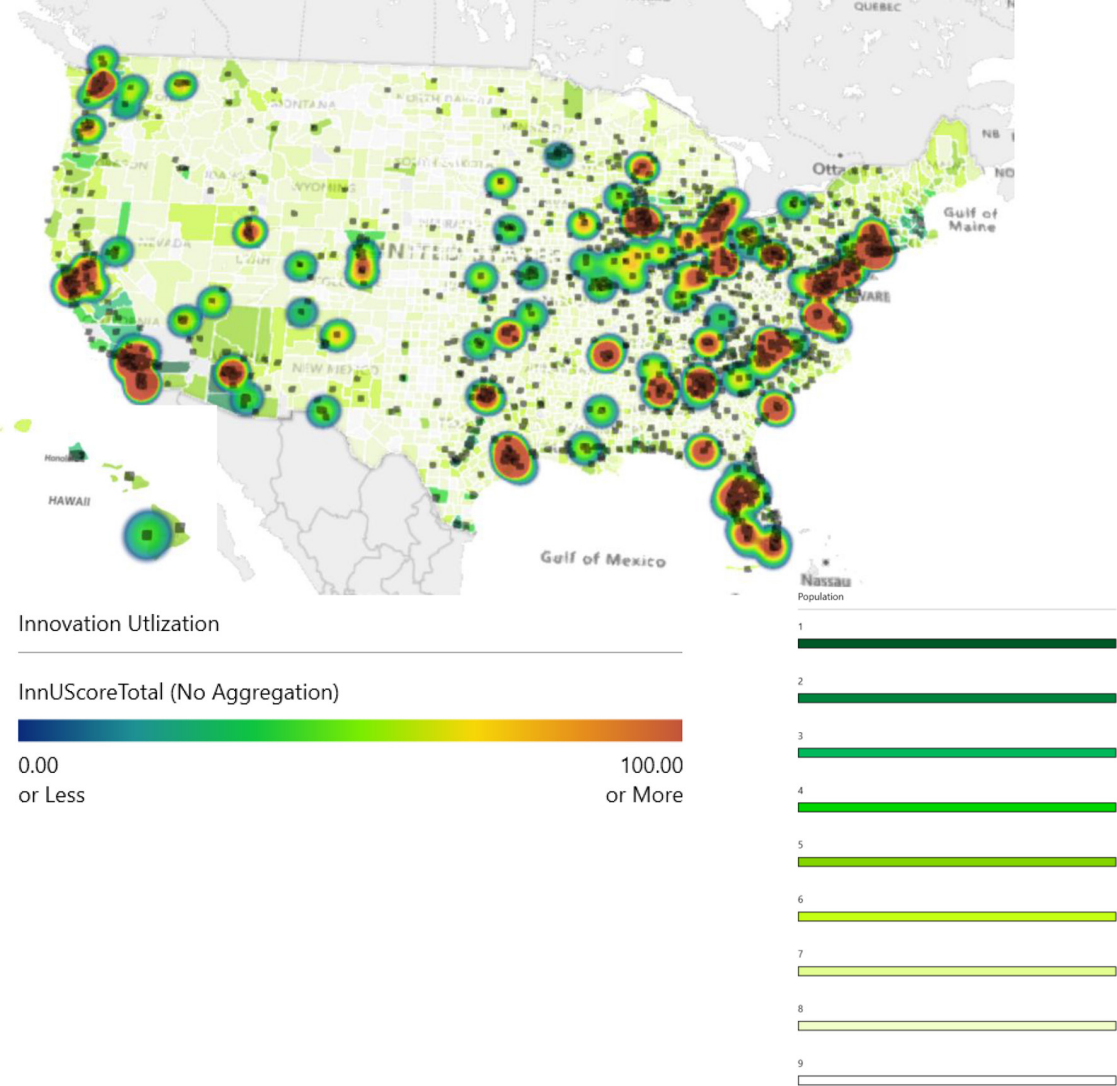
Map of the United States with innovation utilization score.

The results were plotted on a map of the United States for qualitative analysis, as shown in Figure 3. The heat-map represents centers that are more innovative (red) *vs* less innovative (blue). The most innovative centers are in close proximity and in areas with high population density. Conversely, areas with low population density have the lowest innovation score. This qualitative assessment does support the claim that urban centers provide more innovative treatments, despite the absence of large effect sizes and statistical significance.

There was a positive significant correlation between innovation utilization and participant appreciation (*r_s_* = 0.224, *p* = 0.002) and motivation (*r_s_* = 0.215, *p* = 0.003). Both correlations are of small to medium effect size class, according to Cohen’s criteria.^11^ Results for number of meetings attended were not statistically significant.

Bivariate analysis results were not statistically significant for gender, opinion leadership, education and residency status. Bivariate analysis results were not statistically significant for age, DABR status, organizational structure and group size.

At the open-ended feedback section, 63% of the comments were on safety of innovations, which was unexpected by the authors, as all the innovations investigated in the survey were approved by the United States Food and Drug Administration and widely clinically available, *i.e*. not experimental. The comments focused on the theme of safely implementing innovations in the clinic.

## Discussion

The need for a metric of innovation utilization was previously an abstract concept discussed only in theory. This study has now delivered an instrument to quantify innovation in RO. This quantitative instrument, along with qualitative work done by others, will be used to improve innovation utilization in RO. The data collected in this study can serve as a benchmark of the state of innovation today, with plans on how to improve it in the future.

Centers with a university affiliation have a higher mean innovation score than centers without a university affiliation. There are many outcome differences between academic and non-academic centers. While the difference in innovation score is likely not the only factor contributing to outcome differences, it is a factor that needs to be incorporated in future models. The results of this study are in congruence with similar studies performed in the United States.^12^ It is interesting to note that the two categories with statistically significant results are patient treatment and workflow. The parameters affecting the patient treatment innovation score (stereotactic body radiosurgery, stereotactic cranial radiosurgery, robotic radiosurgery, intraoperative radiation therapy, and flattening free beams) are techniques that are only available in newer accelerators, which are in their majority multimillion-dollar investments; possibly out of reach in rural environments. Improving workflows can be a low-risk, high-yield opportunity for many centers lacking the funds for large investments. A curriculum with core and adjunct tools for MPs is currently under development through the Medical Physics Leadership Academy (MPLA) Committee. The MPLA is an AAPM committee under the Professional Council, focusing on collaborative effort to make leadership training available to medical physicists, with particular focus on developing and disseminating training materials that are relevant to the field of Medical Physics and recognized and proven in the field of business management and applied psychology. The 2016 summer school was devoted to the MPLA and leadership development in Medical Physicists, in an effort to make leadership training more formal.^13^ The lack of statistically significant differences in other categories is a positive finding for the industry, as it implies that once centers break through the barrier of purchasing innovative technologies, there are no major differences in utilizing them.

The differences in centers with university affiliation having a higher mean workflow utilization score than centers without a university affiliationis interesting because organizational innovation has not previously been studied in RO in the United States, as typically publications focus on technological differences.^1^ The results of this study are in congruence with the published results from the Netherlands.^4,14^ The authors concluded that in the Netherlands systematic collaboration between centers and a national registry would be beneficial to improving innovation implementation even further. This conclusion holds true for the United States as well, and can be carried under the aegis of the AAPM.

While differences in patient treatment are relatively easy to explain due to purchasing decision and competition in urban centers, the differences in patient positioning may not be so obvious. Patient positioning is typically decided at the time of simulation and is the primary responsibility of the radiation therapists. Historically, there is great variation in MPs involvement in patient positioning and setup reproducibility, with some MPs being very involved, and some MPs being absent in the simulation process.^15^ The introduction of mandatory MP residencies is closing this gap. The increase in hypofractionated treatments has also changed this dynamic, as discussed in the 2014 AAPM summer school on “safely and accurately delivering high precision, hypofractionated treatments” and AAPM reports.^16^ However, there may be discordance of information flowing to the American Society of Radiologic Technologists. Another possible explanation is that in urban centers, patients “shop around” for their treatment, with higher socioeconomic status patients often requesting or demanding certain types of treatment.^17,18^ Frequent examples from the author’s personal experience include prone breast treatments or large full-body immobilization.

There is a positive correlation between innovation utilization and participant appreciation were small, yet the results are in congruence with prior published studies in general and in the healthcare setting.^8^ It is important to note that this is the first time these parameters have been measured for MPs, and results are in agreement with studies done on other healthcare professionals.^9^ Appreciation and motivation are often considered “soft skills” that may be shunned by MPs in leadership positions.^19^ This common misconception is declining since the introduction of the MPLA and the 2016 MPLA summer school. The results of this study will serve to strengthen the base of evidence supporting intra personal skills and clinical performance.

The results of this study have a public health component as well. Inequalities in health are parallel to inequalities in healthcare.^20^ To improve public health further in the 21st century, there needs to be an inclusion of factors outside of traditionally defined healthcare.^21^ Disparities in access to advanced care have an impact on cancer survival. This statement may be considered contradictory by some, but it is well supported by recent literature.^12,22,23^ There is an abundance of differences between centers that may have a causal effect on improved cancer survival^12,22^ and outcomes.^17,23,24^ Innovation is only one of these parameters. This study did not attempt to show causal effects, as this can only be demonstrated by clinical trials.^25^ What this study did demonstrate, however, is that there are indeed differences in innovation accessibility in RO in the United States. The connection between innovation and improved cancer survival has been made by many authors; innovation-based care models are under discussion in reimbursement healthcare reform.^22,26^ Public health is expanding beyond government agency programs to a broader cross-sectoral practice.^21^ RO as a community is in a position to further engage public health aspects that have a collective impact on population health.

The comments on the competitive nature between safety and innovation can only be addressed at the organizational level. Getting MPs to embrace innovation as part of their culture will only be possible if innovation is not considered to be competing with safety. Creating safe innovative programs is a balancing act. Safety and innovation are not contenders, they are building foundations.^27^ The complementary relationship between safety and innovation is being discussed in many other healthcare fields.^28^ The pathway to deteriorating safety would be possible only through poor implementation. This circles back to workflow innovation and it is an area where the AAPM can lead in changing this narrative with initiatives such as the increasing number of Medical Physics practice Guidelines and risk analysis methods described in Task Group 100.^29^

The presented study has limitations. Due to the study design, there was a possible selection bias. The study may not have reached some MPs, especially those who practice in rural areas. Since there are no publicly available proportions of MPs per ZIP code, the effect of this limitation is not possible to calculate. Comparison with known proportions of university *vs* non-university centers showed a reasonable degree of agreement, which implies that selection bias was not a significant source of bias in this study. Another possible source of, information bias, could also have influenced the results. Unfortunately, there is no way to assess the magnitude of this effect. Both of selection bias and information bias are inherent to the study design.

Additionally, there was a high level of internal consistency, as determined by Cronbach's α of 0.963. This statistic in combination with the face and content validity of the expert panel review leads to the conclusion that the constructs have high reliability. However, there were many assumptions made in the operationalization of constructs. It is possible that not all predictive parameters were included in the study, or operationalized appropriately. Further, the operationalization of constructs may not be transferable outside the United States, thus results should be applied with caution outside of the United States.

Furthermore, there are statistical limitations. The effect sizes used to calculate a priori power were hypothetical and chosen conservatively. Post hoc analysis for university status reveals that based on the detected sample effect size, the power of the study was 0.72. This is slightly smaller than the intended 0.8, yet still within reason. Conversely, the power for the RUCA continuum ranged from 0.52 to 0.88, depending on the model selected. This is because of the selected RUCA continuum and the low response rates from areas closer to the rural end of the spectrum. It is uncertain if the effect sizes measured in this study are true population effect sizes or sample effect sizes, thus results should be interpreted with caution until effect sizes are confirmed by future studies.

Lastly, this was a cross-sectional study, thus the study design does not allow the investigation of temporal relationships and possible causality between the dependent and independent variables. However, the results are congruent with theoretical causal structures used in population health.^30^

## Conclusion

In this study, innovation in RO in the United States was measured for the first time, through the development of a new survey instrument. Rural centers and centers with no academic affiliation are trailing behind in innovation implementation. Motivation and appreciation were shown to be statistically significant personal factors influencing innovation utilization. RO practitioners follow an ethos of “as low as reasonably achievable” every day, making every attempt possible to reduce dose to patients. MPs do this almost subconsciously, as it has been engrained in our training as common sense. If every MP practiced using “as innovative as reasonably achievable” as their mantra, similar to “as low as reasonably achievable,” patients would benefit immensely. This study provides a small but promising step in this direction. Although the exact number of lives saved or extended because of innovations in daily practices in RO may never be known, it is certainly worth it to try to make every treatment as innovative as reasonably achievable.
