# Supplementary material for: Diffusion of innovation in radiation oncology in the United States
Source: BJR Open. 2020 Aug 26;2(1):20200025. doi: 10.1259/bjro.20200025 (PMC7583171; doi:10.1259/bjro.20200025)
Supplement: Supplementary Material 1. [file bjro.20200025.suppl-01.pdf]

Diffusion of Innovation in Radiation Oncology Survey

Informed Consent

Introduction and consent

You are invited to take part in a research study about the Diffusion of Innovation in Radiation Oncology (RO). This form is part of the “informed consent” to allow you to understand this study before deciding to take part. This study is being conducted by a researcher named xxxx xxxx, who is a doctoral student at xxxx.

Background Information

The purpose of this study is to investigate the accessibility of innovative services in RO in the United States and assess possible diffusion patterns.

Procedures

If you agree to be in this study, you will be asked to complete online approximately 40 unique questions about how you use innovative technology in your clinic, for example, surface guidance, flattening free beams etc and the barriers you face in using them. This online survey will take approximately five to ten minutes of your time.

Voluntary Nature of the Study

This study is voluntary. You may stop at any time.

Risks and Benefits of Being in the Study

There are minimal risks by participating in this study. You will be asked about how you use innovative technologies you may have in your clinic, in addition to some basic demographic information. These questions may make you think about if you use innovation enough and possibly make you uncomfortable. “Evidenced-based decision making” is a frequently used catchphrase, yet there is a vast disconnect between strong scientific support and diffuse clinical use. Medical Physicists are the "scientists in the room" and their role in the diffusion of new products and practices across the modern RO clinic in the United States is critical. Measuring the diffusion of innovation in RO will pave the way to improve it.

Payment

At the conclusion of the study, you can be compensated for your time with a $10 Amazon gift card. To enter, you will be asked for your email. You may decline by leaving the entry blank and maintain full anonymity. If you choose to receive the gift card, your email will be confidential, as the email address provided will be excluded from the final aggregate dataset.

Privacy

Reports coming out of this study will not share the identities of individual participants. Details that might identify participants, such as location of the study, will also not be shared. IP addresses will not be collected. The researcher will not use your personal information for any purpose outside of this research project. Data will be kept secure and for a period of at least 5 years, as required by the university.

Contacts and Questions

If you have questions later, you may contact the researcher via email at xxxx or the university's Research Participant Advocate at xxx-xxx-xxxx. xxxx University's approval number for this study is 04-24-19-0603259 and it expires on April 23, 2020.

You may wish to print or save this consent form for your records.

Obtaining Your Consent

If you feel you understand the study well enough to make a decision about it, please indicate your consent by clicking "Next" below.

Diffusion of Innovation in Radiation Oncology Survey

Organization Description

First, tell us a few things about the organization you practice in.

1. Does the Radiation Oncology practice you are responding for has a university or university hospital affiliation?

- ◯ Yes, there is a university or university hospital affiliation.
- ◯ No, there is no university or university hospital affiliation.

2. In what ZIP code is the practice located? (enter 5-digit ZIP code; for example, 22314). Answers will be used strictly for geocoding purposes.

[ ]

Diffusion of Innovation in Radiation Oncology Survey

Organization products and technologies used for patient positioning and monitoring

Are any of the following products and technologies used in your department? If yes, to what degree are they used? Use the slider to respond. What barriers do you face for each category?

Category: Patient positioning and monitoring.

3. Surface guidance

Don't have	Sometimes on Some patients	On all applicable patients

4. Respiratory Gating

Don't have	Sometimes on some patients	On all applicable patients

5. Breath Hold

Don't have	Sometimes on some patients	On all applicable patients

What barriers do you face in the implementation of innovation on patient positioning and monitoring techniques?

6. Lack of evidence and publications on the relative advantage of the innovation

0 100

7. Complexity of the innovation

0 100

8. Lack of time to implement, staffing constraints, and emphasis on clinical productivity

0 100

9. Lack of training and support

0 100

10. Lack of interest from others, no supporting organizational culture

0 100

11. Lack of inter-operability with existing technology and practices

0 100

12. Lack of reimbursement

0 100

Diffusion of Innovation in Radiation Oncology Survey

Organization products and technologies used for patient treatment

Are any of the following products and technologies used in your department? If yes, to what degree are they used? Use the slider to respond. What barriers do you face for each category?

Category: Patient Treatment

13. Stereotactic Body Radiosurgery

Don't have | Sometimes on Some patients | On all applicable patients

14. Stereotactic Cranial Radiosurgery

Don't have | Sometimes on some patients | On all applicable patients

15. Robotic Therapy

Don't have	Sometimes on some patients	On all applicable patients

16. Intra-Operative Radiation Therapy

Don't have	Sometimes on some patients	On all applicable patients

17. Flattening Filter Free beams

Don't have	Sometimes on some patients	On all applicable patients

What barriers do you face in the implementation of innovation on patient treatment techniques?

18. Lack of evidence and publications on the relative advantage of the innovation

0 100

19. Complexity of the innovation

0 100

20. Lack of time to implement, staffing constraints, and emphasis on clinical productivity

0 100

21. Lack of training and support

0 100

22. Lack of interest from others, no supporting organizational culture

0 100

23. Lack of inter-operability with existing technology and practices

0 100

24. Lack of reimbursement

0 100

Diffusion of Innovation in Radiation Oncology Survey

Organization products and technologies used for treatment planning

Are any of the following products and technologies used in your department? If yes, to what degree are they used? Use the slider to respond. What barriers do you face for each category?

Category: Treatment planning

25. Automatic/knowledge-based contouring

Don't have | Sometimes on Some patients | On all applicable patients

26. Deformable Image Registration

Don't have | Sometimes on some patients | On all applicable patients

27. Automatic/knowledge-based planning

Don't have | Sometimes on some patients | On all applicable patients

28. Adaptive planning

Don't have | Sometimes on some patients | On all applicable patients

What barriers do you face in the implementation of innovation on treatment planning?

29. Lack of evidence and publications on the relative advantage of the innovation

0 | 100

30. Complexity of the innovation

0 | 100

31. Lack of time to implement, staffing constraints, and emphasis on clinical productivity

0 | 100

32. Lack of training and support

0 | 100

33. Lack of interest from others, no supporting organizational culture

0 | 100

34. Lack of inter-operability with existing technology and practices

0 100

35. Lack of reimbursement

0 100

Diffusion of Innovation in Radiation Oncology Survey

Organization products and technologies used for quality assurance

Are any of the following products and technologies used in your department? If yes, to what degree are they used? Use the slider to respond. What barriers do you face for each category?

Category: Quality Assurance

36. Portal Dosimetry for linear accelerator QA

Don't have Sometimes Use all the time

37. QA trending and statistical process control

Don't have Sometimes Use all the time

38. Automated machine QA

Don't have Sometimes Use all the time

39. Automated plan checks

Don't have Sometimes Use all the time

What barriers do you face in the implementation of innovation on quality assurance?

40. Lack of evidence and publications on the relative advantage of the innovation

0 100

41. Complexity of the innovation

0 100

42. Lack of time to implement, staffing constraints, and emphasis on clinical productivity

0 100

43. Lack of training and support

0 100

44. Lack of interest from others, no supporting organizational culture

0 100

45. Lack of inter-operability with existing technology and practices

0 100

46. Lack of reimbursement

0 100

Organization products and technologies used for workflow

Are any of the following products and technologies used in your department? If yes, to what degree are they used? Use the slider to respond. What barriers do you face for each category?

Category: Workflow

47. Does your clinic participate in clinical trials

Don't participate | Sometimes on some patients | On all applicable patients

48. Does your clinic develop new practices for organizing procedures (for example re-designing workflows to be lean, quality management etc)

0 | Sometimes | 100

49. Does your clinic develop new methods for organizing work responsibilities and decision making (for example new training systems etc)

0 | Sometimes | 100

50. Does your clinic develop new methods of organizing external relationships with other organizations or public institutions (for example alliances for first use of an innovation, partnerships, outsourcing or sub-contracting innovations)

0 | Sometimes | 100

What barriers do you face in the implementation of innovation on workflow?

51. Lack of evidence and publications on the relative advantage of the innovation

0 | 100

52. Complexity of the innovation

0 100

53. Lack of time to implement, staffing constraints, and emphasis on clinical productivity

0 100

54. Lack of training and support

0 100

55. Lack of interest from others, no supporting organizational culture

0 100

56. Lack of inter-operability with existing technology and practices

0 100

57. Lack of reimbursement

0 100

Diffusion of Innovation in Radiation Oncology Survey

58. What is your gender?

- [ ] Male
- [ ] Female
- [ ] Other (please specify)

[ ]

59. Please enter your age

[ ]

60. What is the highest degree you have received?

- ○ Master’s degree
- ○ Doctoral degree
- ○ Other (please specify)

[ ]

61. Have you completed a Medical Physics Residency?

- ○ No, I began my practice before the residency mandate.
- ○ No, I was not accepted in one
- ○ Yes, I successfully completed a Medical Physics Residency

62. Are you certified by the American Board of Radiology?

- ○ Yes, I am a diplomate of the American Board of Radiology
- ○ Not yet, I have only passed Part I
- ○ Not yet, I have only passed Part II
- ○ No, I do not hold any certification
- ○ No, but I hold a different certification

63. How many national meetings have you attended in the past 10 years? Please enter a number. A full list of AAPM and ASTRO meetings is shown below.

AAPM Annual 2009 in Anaheim, California

AAPM Annual 2010 in Philadelphia, Philadelphia

AAPM Annual 2011 in Vancouver, BC

AAPM Annual 2012 in Charlotte, North Carolina

AAPM Annual 2013 in Indianapolis, Indiana

AAPM Annual 2014 in Austin, Texas

AAPM Annual 2015 in Anaheim, California

AAPM Annual 2016 in Washington, District of Columbia

AAPM Annual 2017 in Denver, Colorado

AAPM Annual 2018 in Nashville, Tennessee

AAPM Spring Clinical 2009 in Virginia Beach, Virginia

AAPM Spring Clinical 2010 in San Antonio, Texas

AAPM Spring Clinical 2011 in Chattanooga, Tennessee

AAPM Spring Clinical 2012 in Dallas, Texas

AAPM Spring Clinical 2013 in Phoenix, Arizona

AAPM Spring Clinical 2014 in Denver, Colorado

AAPM Spring Clinical 2015 in Denver, Colorado

AAPM Spring Clinical 2016 in Salt Lake City, UT

AAPM Spring Clinical 2017 in New Orleans, Louisiana

AAPM Spring Clinical 2018 on Las Vegas, Nevada

ASTRO Annual 2009 in Chicago, Illinois

ASTRO Annual 2010 in San Diego, California

ASTRO Annual 2011 in Miami, Florida

ASTRO Annual 2012 in Boston, Massachusetts

ASTRO Annual 2013 in Atlanta, Georgia

ASTRO Annual 2014 in San Francisco, California

ASTRO Annual 2014 in San Francisco, California

ASTRO Annual 2015 in San Antonio, Texas

ASTRO Annual 2016 in Boston, Massachusetts

ASTRO Annual 2017 in San Diego, California

ASTRO Annual 2018 in San Diego, California

ASTRO Annual 2018 in San Antonio, Texas

AAPM Spring Clinical Meeting 2019 in Orlando, Florida

64. Who do you report to?

- ○ Another Physicist (e.g. Chief Physicist / Physics Director/ Physics Chair)
- ○ A Radiation Oncologist (e.g. Program Director / Department Chair)
- ○ An Administrator (e.g. Manager / Director)
- ○ A Vice President
- ○ Other (please specify)

65. How many Physicists practice in the same location, including yourself?

- ○ I am the only one
- ○ 2-3
- ○ 3-5
- ○ 5-10
- ○ >10
- ○ Don’t know

66. Have you ever volunteered in AAPM committees or leadership?

- ○ Yes
- ○ No

67. Do you feel appreciated at work?

0 100

68. Do you feel motivated at work?

0 100

Diffusion of Innovation in Radiation Oncology Survey

Final Thoughts

Please share any final thoughts you may have on innovation in Radiation Oncology

69. Final Thoughts

Diffusion of Innovation in Radiation Oncology Survey

70. If you would like to receive a $10 Amazon gift card, please enter your email. If not, please click next.
