# Supplementary material for: Diffusion of innovation in radiation oncology in the United States
Source: BJR Open. 2020 Aug 26;2(1):20200025. doi: 10.1259/bjro.20200025 (PMC7583171; doi:10.1259/bjro.20200025)
Supplement: Supplementary Material 2. [file bjro.20200025.suppl-02.pdf]

Appendix B: 2013 Rural-Urban Continuum Codes

	Code	Description
Metropolitan	1	Counties in metro areas of 1 million population or more
	2	Counties in metro areas of 250,000 to 1 million population
	3	Counties in metro areas of fewer than 250,000 population
Non metropolitan	4	Urban population of 20,000 or more, adjacent to a metro area
	5	Urban population of 20,000 or more, not adjacent to a metro area
	6	Urban population of 2,500 to 19,999, adjacent to a metro area
	7	Urban population of 2,500 to 19,999, not adjacent to a metro area
	8	Rural or less than 2,500 urban population, adjacent to a metro area
	9	Rural or less than 2,500 urban population, not adjacent to a metro area
